# Realm of Thermoalkaline Lipases in Bioprocess Commodities

**DOI:** 10.1155/2018/5659683

**Published:** 2018-02-14

**Authors:** Ahmad Firdaus B. Lajis

**Affiliations:** ^1^Department of Bioprocess Technology, Faculty of Biotechnology and Biomolecular Sciences, Universiti Putra Malaysia, 43400 Serdang, Malaysia; ^2^Bioprocessing and Biomanufacturing Research Center, Faculty of Biotechnology and Biomolecular Sciences, Universiti Putra Malaysia, 43400 Serdang, Malaysia

## Abstract

For decades, microbial lipases are notably used as biocatalysts and efficiently catalyze various processes in many important industries. Biocatalysts are less corrosive to industrial equipment and due to their substrate specificity and regioselectivity they produced less harmful waste which promotes environmental sustainability. At present, thermostable and alkaline tolerant lipases have gained enormous interest as biocatalyst due to their stability and robustness under high temperature and alkaline environment operation. Several characteristics of the thermostable and alkaline tolerant lipases are discussed. Their molecular weight and resistance towards a range of temperature, pH, metal, and surfactants are compared. Their industrial applications in biodiesel, biodetergents, biodegreasing, and other types of bioconversions are also described. This review also discusses the advance of fermentation process for thermostable and alkaline tolerant lipases production focusing on the process development in microorganism selection and strain improvement, culture medium optimization via several optimization techniques (i.e., one-factor-at-a-time, surface response methodology, and artificial neural network), and other fermentation parameters (i.e., inoculums size, temperature, pH, agitation rate, dissolved oxygen tension (DOT), and aeration rate). Two common fermentation techniques for thermostable and alkaline tolerant lipases production which are solid-state and submerged fermentation methods are compared and discussed. Recent optimization approaches using evolutionary algorithms (i.e., Genetic Algorithm, Differential Evolution, and Particle Swarm Optimization) are also highlighted in this article.

## 1. Introduction

Lipases are lipolytic enzymes, triacylglycerol acylhydrolases (EC 3.1.1.3) that catalyze the hydrolysis of triacylglycerols to glycerol and free fatty acids [1]. Lipases are ubiquitous and versatile in which they can act as hydrolases or synthases and, like any other biocatalysts, they have the ability to catalyze reactions under various conditions with a very high degree of substrate specificity [1–3]. Hence, lipases reduce the formation of by-products from “reaction” process which is one of few factors for their green and environmental friendly properties [1, 2]. For decades, microbial lipases were remarkably used as biocatalyst and their market values in various fields and industries such as food (i.e., aroma), agriculture, cosmetics (i.e., esters), medicine, pharmaceutical (i.e., drugs), detergent (fat/oil removal), fat-processing (i.e., dairy), oleochemical (biodiesel), leather, paper (pitch/sap removal), and textile are increasingly growing [1–8]. Previous study indicates that demands for lipases as biocatalysts are significant, and report launched by the Freedonia Group in 2014 showed that the world demand for lipases is projected to increase 6.2% annually to US$ 345 million in 2017 [1]. To date, lipases are placed at the top third rank behind proteases and amylases and lipases annual market is expected to reach about 590.5 million dollars by 2020 [4].

Lipases synthesized by microbes play important roles as natural functional proteins in which their type and function in bacteria, actinomycetes, and fungi may differ mainly depending on its constitutive genetic makeup and biological evolution in order to optimize their behavioural condition in complex environment under normal-stress condition as well as for their living and survival [8–12]. Lipases show interfacial activation, which is due to the existence of a hydrophobic lid in most lipases. It covers the active center in the inactive closed conformation of the lipases [13]. In its open conformation form, the active center is accessible for the substrates [14, 15]. The change between closed to open forms occurs in the occurrence of a substrate emulsion or a lipid interphase that opens the lid, as a result of which the lipases are activated [13]. Several instrumental techniques for structural analysis such as X-ray crystallographies, circular dichroism, Fourier transform infrared (FTIR), electrospray ionization (ESI), and matrix-assisted laser desorption/ionization (MALDI) have been performed to elucidate the molecular structure of lipases. Three-dimensional (3D) structures of many lipases share similar *α*/*β* hydrolase fold and have the same catalytic mechanism in which active site is formed by a catalytic triad of serine (Ser), aspartatic acid (Asp)/glutamic acid (Glu), and histidine (His) [14, 16]. Lipases also share a consensus pentapeptide sequence of Gly-X-Ser-X-Gly motif, whereby X may be any amino acid residue [14]. For instance, X-ray crystallography analysis of protein structure-function relationships of* Bacillus* sp. L2 lipase revealed that topological organization of *α*/*β*-hydrolase fold consisting of 11 *β*-strands, 13 *α*-helices, serine-113, histidine-358, and aspartate-317 with single Ca^2+^ and Zn^2+^ was found in this lipase molecule [17]. Recently, amino acid sequence of lipase from* S. arlettae* JPBW-1 was characterized using MALDI-TOF-MS analysis and comparative modelling approach was performed via ROBETTA server to postulate a structure-activity relationship of the lipase [16]. In one study, it was found that TA lipase from* Cohnella* sp. A01 was structurally determined to be 37.5%  *α*-helix, 12.8%  *β*-sheet, 22.7%  *β*-turn, and 27% random coil [18]. Although several lipases have been industrially produced, the discovered lipases are not tolerant to high temperature and alkaline environment, thus easily losing their 3D conformational structure at certain period of time [1–3]. Moreover, improvement of lipases stabilities via lipase modification and immobilization also leads to some drawbacks such as high cost of production and time-consumption and some strategy is subjected to “trial and error” [13]. In some applications, immobilized lipase (heterogeneous form) is not preferred; instead free lipases are more suitable for some functions such as in laundry industry [19, 20].

To date, microbial lipases which are stable at high temperature and resistance to alkaline pH known as thermoalkaline (TA) lipases have been reported as new potential biocatalyst. TA lipases may differ from non-TA lipases such as in their primary sequences, molecular weights (MW), optimal pH and temperature, substrate and positional specificities, cofactors, and cellular locations [1, 2]. TA lipases have been given more attention due to their nature which is thermostable and alkaline tolerant in immobilized or in free lipases form. Although several TA lipases have been discovered, they are still under extensive research particularly in the development of fermentation process. Moreover, details of industrial fermentation techniques of TA lipases production are rarely reviewed and some knowledge owned by producing company or entity has been already patented [1]. This review describes and discusses the characteristics and potential applications of TA lipases, as well as the development of TA lipases production via process fermentation and its optimization approaches.

## 2. Characteristics of TA Lipases

### 2.1. Molecular Weight

The MW of the mesophilic enzymes is about the same to that of thermophilic enzymes with nearly similar 3D structures [11, 68]. Most of the thermophilic enzymes appears as small-size monomer and compacted proteins which may confer a higher thermostability as compared to that of the bulky proteins of mesophilic enzymes [31]. In fact, thermophilic enzymes have about double the number of subunits than mesophilic enzymes where enzymes with small subunits are more thermostable as compared to large enzyme with no subunits [68]. Several studies have reported that the MW of polypeptide chain of TA lipases can range from 21 to 67 kDa. This refers to molecular weight of the TA lipases from* B. stearothermophilus, A. cinnamomea* BCRC 35396,* Geotrichum candidum*,* T. atroviride* LipB,* T. atroviride* LipA,* Acinetobacter *sp. AU07,* Microbacterium* sp.,* T. thermophilus*,* Bacillus* sp. RSJ-1,* B. thermoleovorans* ID-1,* Bacillus LBN2, Cohnella* sp. A01,* B. licheniformis* Ht7, and* B. sonorensis* 4R which were 67.0 kDa, 60.0 kDa, 59.0 kDa, 57.0 kDa, 50.0 kDa, 45.0 kDa, 40.0 kDa, 39.0 kDa, 37 kDa, 34.0 kDa, 33.0 kDa, 29.5 kDa, 22.0 kDa, and 21.87 kDa, respectively, as determined using sodium dodecyl sulfate- (SDS-) polyacrylamide gel electrophoresis (PAGE) [4, 9, 11, 18, 23, 26, 27, 29–33, 54, 69–71].

### 2.2. Temperature and pH

Thermophilic lipases are usually optimally active between 60 and 80°C and some lipases are relatively thermostable with optimal active temperature of 40 to 60°C. For instance, TA lipase from* Bacillus thermoleovorans* ID-1, isolated from hot springs in Indonesia, had an optimal activity at 70–75°C and pH 7.5 [31]. TA lipase ID-1 exhibited 50% of its original activity after incubation at 60°C (for 60 min) and at 70°C (for 30 min) and its catalytic function was activated in the occurrence of Ca^2+^ or Zn^2+^ [31]. On the other hand, TA lipase from* Bacillus* sp. LBN2 had optimal activity of 60°C and pH 10 which was slightly different as compared to TA lipase ID-1 [9]. It is well-accepted that enzyme resistant to thermodenaturation is also resistant to extreme pH [68]. Thermostable lipase from* Geotrichum candidum* showed stability at varying range of pH (5–12) and thermostability (15–65°C) [71]. The lipase LBN2 was found to be stable in the pH range of 8–11 and retained 90% activity at 60°C for 1 h [9]. TA lipase 3646 from the* Cohnella* sp. A01 expressed in* E. coli* BL21 (DE3) exhibited maximum activity at 70°C and pH 8.5 [18]. The TA lipase 3646 was also highly stable at the pH range of 8.5 to 10.0 for 180 min [18]. On the other hand,* T. atroviride* 676 lipase retained 78.9% of its original catalytic activity at 65°C and approximately 98% of its original activity at abroad range of pH values from 3.0 to 8.0 [33]. TA lipase from both* Microbacterium* sp. and* Bacillus* sp. RSJ-1 exhibited maximal hydrolytic activity at a temperature of 50°C and a pH of about 8.5 [23, 30]. Moreover, TA lipase RSJ-1 was very stable at 50°C for 60 min and retained 90% of its initial activity for 120 min [30]. However, its half-life (*t*_1/2_) at temperature ranging from 55°C to 75°C was decreased from 240 min to 30 min, respectively [30]. The TA lipase RSJ-1 was also highly stable in a pH range of 8.0 to 9.0 for 120 min [30]. Similarly, TA lipase from* Acinetobacter* sp. with sequence similar to GDSL family of lipases had an optimum temperature and pH of 50°C and 8.0, respectively [27]. The TA lipase from* T. coremiiforme* V3 which was cloned into plasmid pPICZ*α*A and overexpressed in* P. pastoris* X33 showed temperature tolerance at 60°C and retained about 45% of lipase activity at 70°C after 60 min [72]. In contrast, lipase from* T. harzianum* IDM14D had a relatively low thermostability (40°C) as compared to previously stated lipases [73]. The lipase IDM14D was stable at a pH range of 8.0–10.0 with an optimum enzymatic activity at pH 8.5 for 60 min [73]. The lipase from* A. cinnamomea* BCRC 35396 was found to be stable in pH 7–10 with optimum catalytic activity at pH 8.0 but had a very low activity at pH more than 10 [11]. The lipase BCRC35396 activity was also significantly stable at temperature range of 25–60°C, with maximal activity noted at 45°C [11]. Thermostability of TA lipase from* B. sonorensis* 4R was significant at 80°C due to its hyperthermophilic nature. This TA lipase 4R had a decreasing half-life (*t*_1/2_) pattern at temperature ranging from 80°C to 120°C for 150 min to 50 min, respectively [4, 45]. However, TA lipase 4R was highly activated and stabilized by the presence of Mg^2+^ which prolonged its *t*_1/2_ values at 80°C from 150 min to 180 min [4]. TA Lipase 4R was also highly active at pH 9.0 for 160 min [4]. On the other hand, TA lipase from* T. thermophilus* expressed in* P. pastoris* worked efficiently at pH range from 8.9 to 10.5 and showed its maximum activity at pH 9.5 [29]. TA lipase from* T. thermophilus* still possessed 75% of its catalytic activity after treatment at pH 11 for one hour and at temperature of 40°C to 70°C, with an optimal temperature for the reaction at 60°C [29]. High thermal stability was observed for TA lipase from* T. thermophilus*, where more than 70% of its original activity was remained after 60 min treatment at temperature up to 80°C [29]. Very high temperature stability was also reported for LipA and LipB from* Thermosyntropha lipolytica* which were examples of few lipases that had a maximal catalytic activity at a very high temperature (96°C) [15, 39]. LipA and LipB at 100°C and pH 8 also retained 50% of its original activity after 120 to 360 min of incubation period [15].

### 2.3. Salts, Metals and Inhibitors

Most TA lipases were also activated by the presence of several ion salts but greatly inhibited by some other heavy metals and chemicals. It has been reported that salts and metals ions have various effects on TA lipases catalytic activity. For instance, preincubation of TA lipase 4R with inorganic salts (MgSO_4_ and CaSO_4_) stimulated lipase activity by 249.94% and 30.2%, respectively [4, 19]. In contrast, the catalytic activity of TA lipase 4R was greatly reduced in the presence of other inorganic and metal salts [CoCl_2_, CdCl_2_, HgCl_2_, CuCl_2_, and Pb(NO_3_)_2_], enzyme inhibitors [phenylmethylsulfonyl fluoride (PMSF), orlistat], oleic acid, iodine, chelating agent [ethylenediaminetetraacetic acid (EDTA)], and dissociating agent (urea) [4]. Unlike TA lipase 4R, TA lipase from* Cohnella* sp. A01 is not a metalloenzyme as there is no reduction in catalytic activity after treatment with EDTA [18]. On the other hand, the catalytic activities of both LipA and LipB lipases were completely inhibited by 10 mM PMSF and 10 mM EDTA [15]. Further metal ions analysis demonstrated that both LipA and LipB are metalloenzymes containing one Ca^2+^ and one Mn^2+^ ion per monomeric lipase unit [15]. LipA and LipB probably have a structural motif of Ca and Mn-binding site [17]. Moreover, thermal stability of both LipA and LipB is dependent on the addition of 1 mM Mn^2+^ which enhanced their catalytic activities at 96°C by 3-fold and increased the durations of their thermostability up to 4 h at 60°C and 75°C, respectively [15]. Ca^2+^ and Mn^2+^ also enhanced* T. harzianum* IDM14D lipase activity while other metallic ions did not give any effect [73]. TA lipase from* T. thermophilus* was also promoted by Ca^2+^ and inhibited by Zn^2+^ and Cu^2+^ [29]. The TA lipase from thermophilic* Bacillus* sp. RSJ-1 exhibited enhanced lipase activity in the presence of Ba^2+^, Na^+^, Mg^2+^, and Ca^2+^ and was greatly inhibited by Zn^2+^, K^+^, Co^2+^, and Cs^+^ [30]. The EDTA did not affect TA lipase activity from a thermophilic* Bacillus* sp. RSJ-1 [30]. The* Microbacterium* sp. lipase was completely inhibited by PMSF but minimal inhibition was observed when incubated with EDTA and dithiothreitol (DTT) which indicated the presence of metal ions and disulfide-bridge (S-S) [23]. The reason that lipases being activated by some metal ions such as Ca^2+^ and Zn^2+^ was due to the presence of structural motif of Ca-binding EF-hand (i.e., consists of aspartate, glutamine, and glutamate amino acids residues) and Zn^2+^-binding motif (i.e., consists of two histidine and two aspartic acid amino acids residues) which involves ionic and polar dipole interactions [17]. Some metal ions located a bit further from the catalytic amino acid residue and thus are not likely to contribute in the catalytic activity but rather possibly play a role in an improved stability [17].

### 2.4. Surfactants and Organic Solvents

It is well-known that enzyme resistant to thermodenaturation is also resistant to surfactants and organic solvents [68].* Microbacterium* sp. lipase activity was stimulated by Triton X-100 and SDS and inhibited by polysorbate-20 (Tween-20) and polysorbate-80 (Tween-80) [23]. TA lipase from* Acinetobacter* sp. AU07 was inhibited by detergents like SDS and exhibited minimal loss of lipase activity when incubated with hydrogen peroxide (H_2_O_2_), Tween-80, and Triton X-100 [27]. The hydrolytic activity of the TA lipase from* Acinetobacter* sp. AU07 was specific to moderate chain fatty acid esters 4-nitrophenyl palmitate which was used as a substrate [27]. On the other hand, the presence of various oxidants, reductants, and some surfactants reduced TA lipase RSJ-1 activity [30]. Nevertheless, TA lipase from* G. stearothermophilus* was stable towards a variety of surfactants (i.e., Tween-20, Tween-80, Triton–X100, and SDS), anionic detergent (i.e., sodium cholate, sodium taurocholate), and oxidizing agents (i.e., H_2_O_2_, sodium perborate, and sodium hypochlorite) in which more than 82% of its relative activity was retained [26]. Lipases are normally less active in water-free solvents as compared to aqueous solution due to the restricted conformational flexibility and rigidification of enzyme conformations. However, TA lipase from* G. stearothermophilus* is stable in acetone (ACE), acetonitrile (MeCN), methanol (MeOH), ethanol (EtOH), propanol (PROH), hexane (HEX), heptane (HEP), and cyclohexane (CY) up to more than 85% relative activity [26]. On the other hand, lipolytic activity of the* S. aureus* ALA1 lipase was enhanced by diethyl ether (Et2O), whereas nearly 100% of its catalytic activity was retained in 25% (v/v) organic solvents such as ACE, benzene, MeOH, MeCN, PROH, ETOH, or toluene (TOL) [24, 51]. Polar solvents in particular can penetrate into the active site of lipase where the unfolding of proteins occurs due to disturbances to electrostatic charge interactions, hydrophobic interactions, hydrogen bonding, van der Waals forces, and disulfide linkages [2, 27]. Generally, nonpolar solvents possess less ability to strip the essential water off the lipases molecules than polar solvents which is important for their catalytic activity [2, 32, 56].  Table 1 summarized the characteristic of TA lipases.

## 3. The Applications of TA Lipases

General applications of homogeneous and heterogeneous lipases as biocatalysts have been demonstrated in many literatures for the bioconversion and production of many beneficial products such as DAG, esters, and medium- and long-chain triacylglycerol (MLCT) [74–76]. The process for bioconversion of previously mentioned compounds was conducted at relatively low temperature around 40°C [74]. Unlike TA lipases, commonly used lipases faced loss of catalytic activity at high temperature [77]. Moreover, immobilized lipases which were found stable at high temperature were employed for bioconversion of products which eventually add cost to production [74]. However, immobilized lipases were not suitable for single use application such as detergent. Moreover, the cost for immobilization is far expensive as compared to free lipases. In general, the suitability and major applications of TA lipases can be divided, but not limited, into three main industries (Table 2).

### 3.1. Biodiesel

The transesterification of vegetable oils to biodiesel using chemocatalyst (i.e., CuO/Zeolite) has been industrially implemented due to its very high conversion rates and short production time [38, 78–81]. However, this process inherent several drawbacks related to high energy consumption, nonenvironmental friendly reaction, nonbiodegradable catalyst, and harmful by-products and wastes [i.e., acids and heavy metals (Pd, Fe, Cd, Mn, and Zn)] [78]. Therefore, lipases in their heavy metals (Pd, Cd, Fe, Zn, and Mn) form have been implemented to catalyze process for biodiesel production which is environmental friendly (i.e., less hazardous chemicals, lipases reusability, high substrate specificity, less by-products, and lipases biodegradability) and cost-effective (i.e., single step bioconversion of free fatty acids and triglycerides to biodiesel, low substrate molar ratio, less impurities, ease product separation, and recovery) [79, 80]. The discovery of TA lipases with high catalytic activity at high temperature (thermostable) and resistance to various environmental factors inhibition are an advantage for a cost-effective enzymatic process [23, 34]. Heterogeneous lipases in their immobilized form usually enhance their thermal stability, mechanical strength, chemical-physical stability, lipophobic-lipophilic nature, amount of active lipase, renewability, and remaining functionality [82]. Examples of TA lipases that have shown their potential application for biodiesel production are TA lipases from* Geobacillus thermodenitrificans* AV-5,* Microbacterium* sp., and lipZ01 expressed in* Pichia pastoris* GS11 [23, 34, 44]. The purified lipase lipZ01 favourably hydrolysed triacylglycerols contain acyl chain lengths more than 8 carbon atoms, and the bioconversion amount of biodiesel production was almost 92% in a methanolysis process using MeOH and olive oil [44]. Biodiesel was produced via immobilized lipase* Microbacterium* sp. (immobilized on Celite and charcoal support) catalyzed methanolysis process, which enabled a yield of up to 95.1% biodiesel [23]. On the other hand, maximum yield of 94% of biodiesel can be achieved from alcoholysis of Jatropha oil in t-butanol using immobilized* Enterobacter aerogenes* lipase at an oil : MeOH molar ratio of 1 : 4, 50 U of biocatalyst/g of oil, and a t-butanol : oil volume ratio of 0.8 : 1 at 55°C after 48 h of reaction time [47]. Immobilized lipase mediated transesterification of* Simarouba glauca* oil has been successfully carried out under n-hexane solvent system for maximum biodiesel production (91.5% fatty acid methyl esters) which is considered as an economical process and facilitates lipase reusability via sustainable approach [48].

### 3.2. Biodetergent

In detergent, chemocleansing agents may be composed of a mixture of cationic [i.e., dodecyl dimethyl benzyl ammonium chloride (DDBAC)], anionic surfactants [i.e., linear alkylbenzene sulfonates (LAS), sodium dodecylbenzenesulfonate (SDBS), and SDS], nonionic surfactants [i.e., Tweens, Triton, and fatty alcohol polyoxyethylene ether (FAPOE)], and builders (i.e., phosphates, carbonates) [21, 35, 83]. Some of them are nonbiodegradable and toxic to aquatic organisms in which recent study showed that chemocleansing agents such as FAPOE and DDBAC at 1 *μ*g/mL were toxic to aquatic zebrafish larval with defect in their locomotor activity and significant physical deformation [84]. Moreover, auxiliary sewage treatment removes only a small proportion of wastes (i.e., phosphorus) from the influent, while the bigger proportion remaining are released to stream, river, lake, and estuary via wastewater effluent [21, 84].

Therefore, TA lipases serve as an alternative to replace conventional chemocatalysts for laundry cleaning and dishwashing [35, 52, 53]. Studies have proven that incorporation of TA lipases in detergent does not just improve cleaning performance but also promotes environmental sustainability [35]. TA lipases are very stable at high temperature and alkaline environment which is the optimum condition required for maximal cleaning process. Most of them are also compatible with detergents components and resistant to inhibition [22, 24, 25, 50]. TA lipases are also available in their natural free form, soluble, and readily incorporated in liquid-based detergent [35, 83]. TA lipase from* Bacillus* LBN2 was found stable in some surfactants (i.e., ionic and nonionic) and commercial detergents at range of 0.1% to 10% (w/v) [9]. For instance, Triton x-100 and 114 stimulated the TA lipase activity up to 60% due to an increased number of turnovers of lipase by surface active agents [9]. This can be further explained by a decrease in the surface tension of the aqueous phase which maintains the open conformation of lipase and facilitates substrate binding to catalytic site of the lipase that improves its activity [9]. However, TA lipase from* Bacillus* LBN2 was inhibited by Tween-40 and Tween-80 after 1 h of incubation at 50°C [9]. On the other hand, lipase from* B. thermoleovorans *was strongly inhibited by SDS which may be due to the alteration of the active site conformation of the lipase molecular structure that resulted in inhibition and partial inactivation [31, 54]. On the contrary, TA lipase from* Bacillus stearothermophilus* showed its stability and compatibility with commercial detergents [26]. BSK-L lipase was very stable when formulated in detergent and its components such as oxidising agents and surfactants, hence, help to improve detergent effectiveness and washing performance in removing oil stains [22]. In dishwashing experiment, T1 lipase was proven to be stable in most nonionic surfactants and a mixture of sodium carbonate (Na_2_CO_3_) and glycine (Gly) [52, 53].

### 3.3. Biodegreasing

In leather industry, enzymes are necessary to facilitate process and improve leather quality during different stages in leather manufacturing, such as dehairing, curing, soaking, bating, liming, picking, tanning, and degreasing [3]. Enzyme reduces discharges and waste disposed from different stages of leather manufacturing which has been reported to give health hazards [3]. Being thermostable and alkali-tolerant, TA lipases are also suitable as biodegreasing agents in leather industry where they remove pitch and excessive oil from leather, improving the quality of leather [12]. Examples of TA lipases that have manifested their potential application as biodegreasing agent are* Geobacillus thermoleovorans* DA2 and* Cohnella* sp. A01 lipases [54]. Hide and skin contain proteins and fat in the collagen fibers which must be removed prior to tanning process. Lipases specifically degrade fats, unlikely damaging the leather structure [41]. Removing fats via lipases-degreasing process will minimise hazard and environmental pollutions. For leather made of bovine hide and sheepskins, lipases allow improvement of its tensile strength [41].

### 3.4. Natural Flavour and Pharmaceutical

TA lipases also act as biocatalyst for the synthesis of several flavour esters such as isoamyl acetate via transesterification process of short chain carboxylic acids (i.e., vinyl acetate) and alcohols (i.e., isoamyl alcohol) [85, 86]. Flavour esters give a “Natural” taste, odour, and smell for prospect application in food commodity. For example, immobilized lipase-mediated transesterification of isoamyl acetate and other flavour esters under solvent and solvent-free environment is considered as economical biotechnological approach for flavour ester production via continuous mode of operation [85, 86]. A substantially high isoamyl acetate production (95%) was obtained via enzymatic synthesis of isoamyl alcohol with vinyl acetate using immobilized* Rhizopus oryzae* NRRL 3562 lipase at 8 h reaction time [86].

In the application of TA lipases for pharmaceutical commodity, immobilized lipase from* Candida rugosa* has also been used to synthesize lovastatin, a drug which reduces serum cholesterol levels [41]. Lipases may be used as digestive aids and as the activators of Tumor Necrosis Factor (TNF) to treat malignant tumors [41]. The production of fatty alkanol-amides in a solvent-free enzymatic process via amino-lysis of linoleyl ethyl ester with several amino-alcohols leads to the development of chemoselective synthesis of new active molecules for cutaneous application [87].* Candida antarctica* lipase B has been used for by the synthesis of the serotonin reuptake inhibitor (S)-(+)-cericlamine [88].

## 4. Source of TA Lipases

### 4.1. Wild Strains

Although lipases are ubiquitous, the organisms producing TA lipases are rarely isolated. The nature's genetic reservoir which is the main sources for TA lipases could be identified via functional screening or from the DNA extracted from previously unknown organisms using bioinformatic [17]. Mostly, thermophilic microorganisms are very rich and important sources for TA lipases and they are normally isolated from the soil of areas with special conditions. For example, several extracellular TA lipases have been identified from Gram positive bacterial strains such as* Geobacillus thermoleovorans* (i.e., strain DA2, ID-1),* Geobacillus thermodenitrificans* (i.e., IBRL-nra, AV-5),* B. stearothermophilus, Cohnella thermotolerans*, and* Thermosyntropha lipolytica* DSM 11003 [12, 26, 54]. These thermophilic organisms grow optimally at temperature between 50 and 80°C.

Some other extracellular TA lipases have been also identified from mesophiles. For instance* Aeribacillus* sp. SSL096201,* Bacillus* sp. LBN 2,* B. sonorensis* 4R,* B. licheniformis* Ht7,* B. pseudofirmus, B. odyssey*,* B. pumilus* HF544325, and* Microbacterium* sp. [4, 9, 23, 32, 59, 60]. Gram negative bacterial strains also produce TA lipases such as* Acinetobacter* sp. (i.e., strain AU07, BK44),* Pseudomonas* sp., and* Enterobacter* sp. Bn12 [21, 27, 61]. On the other hand, fungus strains producing TA lipases such as* Talaromyces thermophilus*,* Trichoderma atroviride* 676,* Curvularia* sp. DHE 5, and* Antrodia cinnamomea* BCRC 35396 have been reported [11, 20, 29, 33, 57]. Most of TA producing strains were isolated from several places like desert ecosystem, hot spring, oil-contaminated soil, soil samples of olive oil mill, mushroom spring, alkaline hot spring, pulp and paper mill effluent sludge, alkaline lake, and soil of slaughter house [6, 39, 80].  Table 3 summarized the wild-type (WT) strain of TA lipases.

On the other hand, extremophiles such as hyperthermophiles are also sources for TA lipases. They grow optimally at high temperatures between 80 and 110°C and some even reached up to 113°C [46, 89]. Only represented by few bacterial and archaeal species, these microorganisms have been isolated from marine and terrestrial hot ecosystems. TA lipases from hyperthermophiles developed a special structure-function relationship of high thermal stability and optimum activity at temperatures greater than 70°C [89]. Some of these enzymes are active and thermostable at temperatures up to 110°C and above [89]. Active at very high temperatures, hyperthermophilic enzymes normally do not work well at temperatures below 40°C [46]. For instance, both* B. sonorensis* 4R and* Bacillus* sp. HT19 produced hyperthermostable alkaline lipases [4, 28].

### 4.2. Mutant Strains

Several TA lipases-producing strains have been improved via mutagenesis to obtain strains capable of producing a substantially high amount of TA lipases and even more stable at higher temperature and alkaline pH as compared to WT strains. For instance, mutant strain* P. cyclopium* has been developed using methods of error-prone PCR-direct evolution and site-directed mutagenesis with an improved thermal stability of alkaline lipases (i.e., L41P, G47I, and PCL) [90]. The mutant lipases exhibited an optimal temperature of 5°C higher than the WT strain and half-life of 7 to 13-fold higher than that of WT strain at 45°C [90]. The mechanism responsible for the thermal stability improvement was elucidated from the lipase structure related to leucine-41-proline and glycine-47-isoleucine that could stabilize the PCL structure via hydrophobic-hydrophilic interactions, helix propensity, and proline substitution. The thermal stability for D311E lipase (70.6°C) was higher than WT T1 lipase (68.5°C) [56]. The stability of T1 lipase from* G. zalihae* strain was improved via insertion of an extra ion pair at the intraloop and the interloop mutant D311E via site-directed mutagenesis [52, 56]. On the other hand, mutant S385E lipase which was developed via amino acid substitution strategy has also been reported. It had an increased duration of thermostability and even higher optimal temperature (80°C in Tris–HCl pH 8) as compared to other mutants and WT lipase (70°C in Gly–NaOH pH 9) [43].

### 4.3. Recombinant Strains

TA lipases production was also improved through recombinant techniques. For example, TA lipase TTL gene from* T. thermophilus* was successfully expressed in* Pichia pastoris* with an enhanced extracellular production [29]. The recombinant plasmid pPIC9K-TTL was constructed by inserting TTL gene into the downstream of AOX1 promoter and *α*-factor, which was then transformed into* P. pastoris* via electroporation, producing more than 200 positive transformants [29]. The TA lipase gene from* T. coremiiforme* V3 was also cloned into plasmid pPICZ*α*A and overexpressed in* P. pastoris* X33 [91]. Another lipase gene like Gene lipZ01 was expressed in* P. pastoris *GS115 to produce 42 U/mL recombinant lipase LipZ01 (50 kDa) with maximum activity at optimal temperature and pH value of 45°C and 8.0, respectively [44].

On the other hand, lipase 3646 gene from* Cohnella* sp. A01 was constructed using pET26 b(+) vector and expressed in* E. coli* BL21 (DE3) in which the purified lipase exhibited maximal activity at 70°C and pH 8.5 [18, 76]. Lip 42 gene from* Bacillus* sp. 42 was also cloned in* E. coli* BL21 (DE3) [92]. Some recombinant proteins produced by* E. coli* have few drawbacks such as intracellularly produced lipases and therefore the production is normally proportional and limited by the number of cells [92, 93]. When it comes to downstream processing, extracellular lipases may be preferred as compared to intracellular lipases due to the tedious cell disruption process in order to release the enzymes. Compared with* E. coli* and* S. cerevisiae*, the methylotrophic* yeast P. pastoris* has several benefits as a host for the biomanufacture of recombinant heterologous enzymes, such as easiness of genetic manipulation, high cell density, elevated levels of productivity, the capability to carry out multifaceted posttranslationally modifications, and very small secretion levels of endogenous proteins [91].

## 5. Biomanufacturing

### 5.1. Inoculums Size

In general, inoculums size can range from 1 to 5% (v/v) for optimum TA lipase production depending on strains and fermentation medium (Table 4). For bacterial strain, inoculums were normally prepared using 6 to 16 h old culture before inoculated into basal medium. For instance, inoculums size of 5% (v/v) of a 12 h old culture of* T. lipolytica* was used to produce extracellular TA lipase. Inoculums size of 5% (v/v) of a 12 to 16 h old culture of recombinant* E*.* coli* BL21 harbouring lip42 gene has been also reported. In response of surface methodology (RSM) optimization study, a very low inoculums size was suggested for the production of lipase from* Acinetobacter* sp. AU07 (0.44%, v/v) [27]. Meanwhile, the production of TA lipase from* B. sonorensis* 4R and* G. thermoleovorans* DA2 was conducted at inoculums size of 5% (v/v) and 4% (v/v), respectively [4, 12]. It has been reported that* Bacillus *sp. LBN2 at inoculums size of 10^4^ cells per mL was inoculated into medium for maximal lipase activity [9]. For fungus strain, inoculums were normally prepared using 2 to 10 d old culture before being inoculated into basal medium. For example,* P. chrysogenum* and* A. flavus* strains were prepared using 10 d old culture with 10^6^ spores per gram dry substrate (gds) in solid state fermentation (SSF) [41, 94]. In another study, 1 × 10^7^ spores per mL of* A. niger *J-1 and* A. terreus* NCFT 4269.10 from a 7 d old culture were used as inoculums [95]. A relatively low inoculums size of 1% (v/v) of filamentous fungi* T. atroviride* 676 has been suggested [6, 33]. It is essential to determine the optimum inoculums size for optimal number of active microbial cells needed for TA lipases production. Large inoculums size not only can cause overproduction of microbial mass and inefficient mass transfer but also is unsuitable for scale up process and production in large bioreactor.

### 5.2. Carbon and Nitrogen Sources

A variety of carbon and nitrogen containing substrates could be used as carbon and nitrogen sources for TA lipases fermentation. The type, amount, and ratio of carbon and nitrogen sources are important for microbial growth and optimal production of TA lipases. The most favourable carbon sources for TA lipase production by various thermophilic microbes are varied. Olive oil containing large percentage of monounsaturated fats was mostly used as carbon source and in some cases as an inducer for lipase production. For instance, TA lipase was optimally produced by* B. licheniformis* Ht7,* A. cinnamomea* BCRC 35396,* B. thermoleovorans* ID-1,* Bacillus* sp. IHI-91, and* T. atroviride* 676 using 1–5% olive oil as carbon source in 24 h to 168 h fermentation [11, 33]. This is potentially due to TA lipase substrate specificity to unsaturated fatty acid. In addition to this, isolate* B. thermoleovorans* ID-1 could also cultivate on several lipid substrates such as oils (i.e., olive, mineral, and soybean oil), emulsifiers (i.e., Tween-20, Tween-40, and Tween-66), and triglycerides (i.e., triolein, tributyrin). Another lipase production by* Bacillus* species like* B. coagulans* BTS-3 and* Staphylococcus aureus* was obtained in 48 h using mustard oil as carbon source, probably due to high monounsaturated fats in this oil [42, 60]. TA lipase was also produced by anaerobic* T. lipolytica* DSM 11003T which was grown in a basal medium containing 0.75% (v/v) yeast extract under nitrogen gas phase (anaerobic condition) [15]. TA lipase production was optimally produced by* G. thermoleovorans* DA2 in a medium containing disaccharide galactose (1%, w/v) and ammonium phosphate ((NH_4_)_3_PO_4_, 0.5%, w/v) at carbon/nitrogen ratio of 2 : 1 [1].

In RSM optimized TA lipase production by* T. atroviride* 676, a very high yield of TA lipase (101.75 U/ml) could be obtained using optimal concentration of olive oil and yeast extract as carbon and nitrogen source, respectively [33]. Organic nitrogen source like yeast extract improved not only TA lipase production by* T. atroviride* 676 but also TA lipase production by* S. maltophilia* and* P. gessardii*, which may be due to the metabolic suitability and effective mass transfer of vitamins to the microbes [33]. The production of TA lipases from* T. atroviride* 676,* G. stearothermophilus,* and* P. gessardii* was less enhanced by addition of inorganic nitrogen sources [i.e., ammonium sulfate, (NH_4_)_2_SO_4_] [26, 33]. In one-factor-at-a-time (OFAT) study, a maximum TA lipase from* G. stearothermophilus* was produced using xylose (C_5_H_10_O_5_) as carbon source, followed by glucose (C_6_H_12_O_6_) and sorbitol (C_6_H_14_O_6_). Other carbon sources such as galactose (C_6_H_12_O_6_,), mannitol (C_6_H_14_O_6_), starch (C_6_H_10_O_5_)_n_, lactose (C_12_H_22_O_11_), and sucrose (C_12_H_22_O_11_) were not suitable for production of TA lipase from* G. stearothermophilus* [26]. Starch concentration above 2% (w/v) reduced the lipase production, which could be attributed to the inhibition at high carbon concentration, viscosity, and low level of dissolved oxygen (DO) [34]. This has been supported by several works on reduction of lipase production by many sugars and complex substrates at higher concentrations [26, 34].

Peptone was found to be the most suitable nitrogen source for TA lipase from* G. stearothermophilus* followed by tryptone, gelatine, skim milk, soy protein, and yeast extract [26]. Olive oil is the best source of oil which can act as an inducer for the production of lipase from* Bacillus stearothermophilus*, followed by nut oil, sesame oil, sunflower oil, Tween-80, Triton-X-100, and Tween-20 [26]. High TA lipase from* B. sonorensis* 4R was obtained when glucose (10 g/L) and polysorbate (Tween-80, 10 mL/L) were used as carbon source under static conditions for 96 h fermentation time [4]. On the other hand, high TA lipase yield by* B. Pumilus* HF544325 can be produced using a mixture of peptone and yeast extract as nitrogen sources and glucose as carbon source [33, 60].* T. harzianum* IDM14D was best cultivated using glucose and peptone as carbon and nitrogen sources, respectively, for maximal lipase production (0.24 U/mL) as compared to glucose and yeast extract mixture (0.15 U/mL) at 30°C for 7 d fermentation time [47].

Moreover, lipases activity of some other fungi, such as* B. cepacia, M. hiemalis*, and* A. wentii*, was also stimulated by addition of glucose into the basal medium [15, 17, 46, 74]. Although TA lipase production from this strain is constitutive, the incorporation of oils in the medium increased its final yield. The yield was greater in a medium containing lipidic substrates such as oils as the carbon sources with an addition of organic nitrogen source, but, sometimes, lipase production was also repressed by polysaturated, long chain fatty acid (LCFA) and esters [17, 46, 74]. Castor oil and sesame oil induced lipase production better as compared to other lipid/oil sources. Castor oil contained about 90% ricinoleic acid (C_18_H_34_O_3_) which induces and promotes lipase production [27]. RSM optimization for lipase production by* Acinetobacter* sp. AU07, suggests a significant increase in lipase yield using castor oil as an inducer (2.3%, v/v) to give the maximum lipase activity of 15.84 U/mL [27]. The chain length specificity of the TA lipase gene from* T. coremiiforme* V3 producing two types of LipA and LipB lipases exhibited an elevated activity with p-nitrophenyl laurate and LCFA glycerides such as trioleate (C18:1) tripalmitin (C16:0) where hydrolysis of ester bonds occurred at 1,3-positions [83]. Lipases show very low activity when substrates are in the monomeric form as lipases are activated at the water-lipid (oil) interface. The activity increases significantly when substrates form emulsions due to interfacial activation. This explains the necessity of emulsions for maximum lipolytic activity for the majority of lipases [42, 83].

### 5.3. Other Minerals

Additional minerals were required for TA lipase stability and to boost TA lipases production. Inorganic salt like MgSO_4_ played a significant role on TA lipase production by* T. atroviride* 676, yielding an enzymatic activity of 101.75 U/ml [33]. TA lipase production by* B. sonorensis* 4R was enhanced with the presence of several ions using inorganic salt broth containing 0.001 to 2 g/L of dipotassium phosphate (K_2_HPO_4_), sodium chloride (NaCl), ammonium sulphate [(NH_4_)_2_SO_4_], manganese chloride (MnCl_2_), calcium carbonate (CaCO_3_), ferric (II) sulfate (FeSO_4_), magnesium sulfate (MgSO_4_), zinc chloride (ZnCl_2_), and calcium sulfate (CaSO_4_) adjusted to pH 9.0 [4]. It was found that lipase stability of* B. sonorensis *4R was enhanced when Mg^2+^ and mannitol were added to the fermentation medium [4].* T. lipolytica* DSM 11003T lipase was grown in a basal medium containing 0.01% to 0.3% (w/v) NaCl, K_2_HPO_4_, Na_2_CO_3_, potassium chloride (KCl), sodium sulphide (Na_2_S·9H_2_O), ammonium chloride (NH_4_Cl), magnesium chloride hexahydrate (MgCl_2_·6H_2_O), calcium chloride dehydrate (CaCl_2_·2H_2_O), sodium bicarbonate (NaHCO_3_), semiessential proteinogenic amino acid, cysteine (C_3_H_7_NO_2_S), and some vitamin and trace elements [15, 39]. Certain metal ions give positive effect to TA lipases synthesis and stability due to the presence of metal binding motif in its structure. Some other minerals such as phosphate ions and NaCl provide ATP synthesis and osmotic balance for cell growth and TA lipases production.

### 5.4. Fermentation Temperature

The optimum growth condition (i.e., temperature, pH, etc.) of microbial fermentation may not necessarily be the best condition for highest lipases production even though studies have shown that microbial growth is proportionally increased with TA lipases production and activity. Temperature regulates and gives an effect to the lipase synthesis at mRNA transcriptional regulation of lipase gene and also translation levels of lipase proteins. This involves several other proteins along the way such as regulatory protein, polymerase (i.e., the expression of gene), and helper protein (i.e., assist in periplasmic lipase folding) [24, 41, 89, 96]. High production of several TA lipases was observed at elevated temperature ranging from 28 to 37°C. It has been reported that TA lipase production was best produced at 30°C by* T. harzianum*,* A. radioresistens*, and* A. calcoaceticus* LP009 [47]. The optimum growth condition and lipase production by* T. atroviride* 676 and* B. Pumilus* were at 28°C and 37°C, respectively [33]. Under optimized condition, high lipase production by* Acinetobacter* sp. AU07 was at temperature of 34°C [27]. Higher temperature for microbes mentioned above was not however favourable for TA lipases production, probably due to denaturation of regulatory peptides and proteins, responsible for lipases production. High temperature may also disturb the stability of cell membranes structure as not all microbes have a heat-tolerant cell membrane [41]. Production of TA lipases at these temperatures (28–37°C) has some advantages such as low energy consumption (i.e., due to moderate temperature). Studies show that optimal temperature for production of TA lipase may not correlate to the temperature of the TA lipases activity, which is normally active and stable at a higher temperature.

Other TA lipases are highly produced at high temperature and this includes TA lipase production by* B. stearothermophilus*, which was maximally produced at 55°C [26]. A very high TA lipase production by* B. sonorensis 4R* was reported at 80°C while* G. thermodenitrificans* AV5 and* Bacillus* sp. LBN2 were obtained at 50°C [4, 9].* B. thermoleovorans* ID-1, on olive oil (1.5%, w/v) as the sole carbon source, grew very rapidly at 65°C with its specific growth rate (*μ*max) of 2.5 per hour and maximal lipase activity of 520 U/L [28].* Bacillus* sp. strain IHI-91 also grew optimally at 65°C on olive oil with a *μ*max of 1.0 per hour and maximum lipase productivity of 340 U/L/h [31]. High TA lipase was significantly produced by* T. lipolytica* DSM 11003T at 60°C [9, 15]. Most of the microbes are thermopiles or hyperthermophiles and capable of withstanding and growing at a very high temperature. High TA lipases at these temperatures are usually related to high kinetic rate and mass transfer rate (MTR) at high temperature [82]. An elevated temperature may influence their secretion, possibly by changing the physical properties of the cell membrane, thus allowing more secretion of extracellular lipases [56].

### 5.5. Agitation Rate

In shake flask culture (SFC), agitation rates between 100 and 250 rotations per min (rpm) have been reported for the production of TA lipases. For instance, TA lipase production by* T. atroviride* 676 at agitation rate of 105 rpm and* G. thermoleovorans* DA2 at agitation rate of 120 rpm can reach a maximum production of 175.20 U/mL and 1021.91 U/mL, respectively [1, 33]. The production of TA lipase* Acinetobacter* sp. AU07 can be optimized at agitation rate of 150 rpm [3, 27].* Bacillus *sp. LBN2 in fermentation medium was agitated at 200 rpm for 48 h to give about the highest TA lipase activity of 16 U/g [9]. The lipase production by* B. pumilus* and* T. harzianum* IDM14D was incubated aerobically at 200 rpm and 150 rpm, respectively, at a temperature of 37°C/30°C for 3 to 7 d on a rotary incubator shaker [60]. RSM optimization for lipase production by* Acinetobacter* sp. AU07 with an agitation rate of 199 rpm gave the maximum lipase activity of 15.84 U/mL [27]. Study of agitation rate in SFC serves as a basis for TA lipases production in bioreactor. Due to geometry difference of shake flask and bioreactor, the effect of agitation rate on lipases production was normally reevaluated in bioreactor. Furthermore, in bioreactor, such as stirred tank bioreactor (STB), agitation rate is controlled by one or more impellers. The effect of different impeller design may give some other effect such as shear stress especially at a very high agitation rate which may damage cells and pulpy state moulds [41].

### 5.6. Fermentation pH

Medium pH is very significant in nutrients absorption and growth of microorganisms, stimulation of lipase production via signaling pathways, and release of extracellular lipases. In SFC, the effect of pH on the production of TA lipases was solely conducted at initial pH of the fermentation medium and pH was not controlled throughout the fermentation time due to small fermentation size. The initial pH of 6.0 was found to be the optimal TA lipase production by* T. atroviride* 676 although the optimal TA lipase activity was at pH 8.0 [33]. TA lipase production was also high at initial alkaline fermentation pH by* T. lipolytica* DSM 11003T (pH 8.2) and* B. sonorensis* 4R (pH 9.0) [4, 19]. The lipase production of* Bacillus *sp. LBN2 was found high at alkaline pH range of 8.0 to 10.0 where the maximal production can be obtained at initial pH 9.0 of the fermentation medium [9]. It was suggested by RSM that a significant increase in lipase production by* Acinetobacter* sp. AU07 could be achieved at fermentation pH 7.8 [27]. The optimum initial pH for maximal production of TA lipases by various microbes is varied maybe due to their nature and habitat where they were isolated. Initial pH of the fermentation medium serves as starting pH for the microbes to grow. Initial pH was usually enough for high production of TA lipases and addition of acid or base to control pH medium at certain value may inhibit growth and production of TA lipases. The microbes will slowly adapt to the changes in fermentation medium as pH drop or rise which explains the reason that medium was only set to an initial pH. In few cases, some TA lipases production can be optimized at controlled pH of the fermentation medium [41].

### 5.7. Aeration Rate and Dissolved Oxygen Tension

Aeration rate and dissolved oxygen tension (DOT) are important parameters as most microbes reported in literatures were aerobes or facultative anaerobes. The influence of aeration rate cannot be studied in SFC but it can be controlled in bioreactor (i.e STB) system using a rotameter or flow meter. In SFC, aeration rate and dissolved oxygen (DO) are solely determined by agitation rate but this is not the case for bioreactor system where the agitation and aeration rate can be set separately and maintained at a desired value [92]. Moreover, DOT in SFC cannot be measured and its effect on fermentation could not be evaluated. However, in bioreactor system, DOT can be monitored using DO probe and controlled throughout the fermentation [93]. In bioreactor system, the production of TA lipases was enhanced at aeration rate of 1.5 to 3.0 L/min in 2 L bioreactor which is equivalent to 1.5 to 3.0 vvm (volume of air under standard conditions per volume of liquid per minute). Several reported literatures showed that DOT controlled at 20% of saturation during production period can give maximum TA lipases production. Fermentation of* Acinetobacter* sp. AU07 in 3 L STB at controlled aeration rate of 1.5 vvm increased lipase production considerably to 48 U/mL as compared to SFC (15.84 U/mL) [27]. It has also been reported that DOT was controlled at 30% of saturation for the production of recombinant TA lipase using* E. coli* BL321 harbouring BTL2 lipase gene (Table 5). These reported studies have demonstrated that the effect of aeration rate and DOT in bioreactor play a major role and responsible for the increase in growth of the aerobes and production of the TA lipases. During growth phase and in a situation where oxygen MTR was limited, moulds cells in the form of mycelia pellet may be prone to autolysis and caused void formation in the interior part of the pellet [41]. The TA lipases production in the nonagitated fermentation culture can also be restricted by MTR where oxygen uptake by the cells was scarce [92, 93].

### 5.8. Bioreactor Design

TA lipases production in SFC has its own limitation due to the fact of its small size and geometrically not scalable. A successful optimization in small scale fermentation condition was needed for further study in pilot and industrial scales for possible industrial-scale production via bioreactor system (Table 6). Conventional and new bioreactors as well as impeller designs were studied and compared in terms of their efficiency and feasibility for large-scale TA lipases production. Microbial TA lipases may be produced in very different types of bioreactors such as STB, packed beds bioreactor (PBB), fluidized beds bioreactor (FBB), basket bioreactor (BP), and tray bioreactor (TB) [92, 97]. TA lipase production by an anaerobic* T. lipolytica* DSM 11003T was conducted in 20 L anaerobe bioreactor under nitrogen gas phase where pH of the fermentation was maintained at 7.6 and 9 to give lipase specific activity of 0.15 U/mg and 0.12 U/mg for 18 h and 21 h fermentation time, respectively [15, 39]. On the other hand, submerged fermentation (SmF) of* Acinetobacter *sp. AU07 in 3 L STB increased growth and lipase production to 3.2 (OD_600_) and 48 U/mL, respectively, as compared to SFC (OD_600_ = 1; 15 U/mL) under similar fermentation conditions (i.e., temperature, pH, inducer, inoculums size, and agitation) in 16 h fermentation time, which explained the correlation of an excellent microbial growth obtained in STB to an increased lipase production [27]. Moreover,* B. cepacia* lipase production (50 U/mL) and its specific activity (160 U/mg) in 14 L STB were also higher as compared to the one conducted in SFC (33 U/mL; 112.5 U/mg) under similar experimental condition [i.e., inoculums size, 3% (v/v); temperature, 45°C; uncontrolled pH] [46]. High lipase production and activity from 14 L STB system could be attributed to better nutrients availability and efficient oxygen mass transfer due to the controlled DOT at 25% of saturation with cascade aeration (up to 4 L/min) and agitation rate (between 300 and 1600 rpm) [46, 88].

In SFC, the recombinant* E. coli* BL321 harbouring BTL2 lipase gene under temperature-inducible kPL promoter enhanced lipase specific activity up to 706 kU/g after temperature-induction (shifting of the temperature from 30°C to 45°C) [93]. Later,* E. coli* BL321 harbouring BTL2 lipase gene was produced in a 2 L STB via fed-batch mode of operation, which resulted in slight increase of lipase specific activity up to 770 kU/g [93]. Slightly higher TA lipase BTL2 production and activity in STB as compared to SFC may result in a better control and heat transfer in STB, in consequence with the improved response of kPL promoter triggered by 45°C induction temperature [46, 93]. Meanwhile, recombinant* P. pastoris* X33 harbouring TA lipase gene from* T. coremiiforme* V3 cloned into plasmid pPICZ*α*A was also studied in STB and SFC [83, 91]. Followed by an induction with MeOH, a maximum V3 lipase production (4 to 5 kU/mL) could be achieved in 5 to 50 L STB via fed-batch mode of operation which was about 27- to 33-fold higher as compared to SFC (0.15 kU/mL) in 7 d fermentation time [91].

Moreover, lipase production by* S. warneri* EX17 was optimized under optimal volumetric oxygen MTR (k_L_a) 38 per hour and at pH 7, via SmF in 2 L batch STB using glycerol (C_3_H_8_O_3_) as a carbon source [82]. Under these conditions, the cell concentration reached its maximal value of 8.0 g/L, and the lipase specific activity reached a very high level, approximately 150 U/g cells, which is about five times higher than that obtained in the SFC after 12 h of cultivation [46, 82].

In SFC, the production of TA lipase from* C. freundii* IIT-BT L139 was optimized via OFAT [34]. A very high TA lipase IIT-BT L139 activity (8.8 U/ml) can be obtained at optimal temperature, pH, carbon, and nitrogen sources of 40°C, 9.0, starch, and peptone-urea, respectively, as evaluated via OFAT [34, 61]. When TA lipase IIT-BT L139 was produced via 1 L and 10 L STB (without pH and DOT controlled during fermentation), the activity was found to increase by 36% (12 U/ml) as compared to SFC [34]. STB is a common bioreactor design used for SmF technique while other types of bioreactors such as PBB and TB are more suitable for SSF technique. For instance, lipase production from fungus strain (i.e.,* Penicillium brevicompactum, Burkholderia* sp.) could be produced optimally in TB at temperature of 30°C, moisture content of 70% (w/v), and carbon source concentration (i.e., olive oil and molasses) of 6.25% (g/g) to provide high lipase activity up to 20 U/g [41, 94]. By means of PBB with molasses as carbon source, air superficial velocities of more than 55 cm/min, and temperatures below 28°C, a maximum lipase activity of 26.4 U/g could be achieved, which were 30% higher than that obtained in TB [94]. The lower optimal temperature found using PBB is probably linked to radial heat gradient built inside the PBB [94].

### 5.9. Solid State and Submerged Fermentation

In industry, cost and simplicity of the production techniques are crucial factors for mass-scales TA production. The feasibility of using various types of fermentation techniques (i.e., SSF, SmF) and modes of operation (i.e., batch, fed-batch, and continuous) on the improvement of TA lipases production have been reported. Nevertheless, when compared to SmF, very few attempts had been made to produce TA lipases using SSF. Generally, SSF is known as a method where fungus and moulds are grown on a solid medium with limited free liquid phase of the culture [41, 97]. TA lipases production by SSF of* Aspergillus* sp. and* Rhizopus* sp. had been reported in several studies. For example, a comparatively high activity of* Aspergillus terreus* lipases (MW of 46.3 kDa, thermostable at 60°C) was obtained in SSF after incubation at 30°C for 96 h fermentation time using palm oil as a substrate probably due to suitable composition and ratio of the fatty acids present in palm oil as compared to oils of the sunflower, almond, coconut, olive, castor, mustard, and sesame [95]. Meanwhile, the thermotolerant* Rhizopus homothallicus* produced more lipases (MW of 29.5 kDa) in SSF (10,700 U/mg) as compared to SmF (8600 U/mg) in which maximal activity occurs at optimal temperature of 30°C in SmF and 40°C in SSF [98]. The thermostability, half-life (*t*_1/2_) at 50°C in SmF (0.44 h) was shorter than in SSF (0.72 h) which may be due to different lipases produced from the culture medium as the effect from different fermentation techniques [98]. The lipase production (49.37 U/g) by* Aspergillus niger* AS-02 using sheanut cake under SSF with addition of 1.0% (v/w) Tween-80, 0.35% (w/w), (NH_4_)_2_SO_4_, and 0.40% (w/w) Na_2_HPO_4_ [99]. However, the fermentation parameters such as temperature, pH, and DOT are difficult to control in the SSF. In contrast to SmF, the SSF process occurs in very low water content and thus the lipases are strongly adsorbed on the insoluble biomass, which later require an efficient extraction process and lipase recovery. In an economic analysis of the production of* Penicillium restrictum* lipase in SmF and SSF, it was suggested that SSF techniques offers greater advantage over SmF in terms of raw material, total capital investment, unitary product, cost product selling price, and profitability [41, 100]

Most reports on TA lipases production existing in the literatures are associated with SmF. It is widely used for high performance production of many biomolecules and enzymes including TA lipases. SmF cultures have some advantages over SSF, such as higher homogeneity and more facility to control parameters such as temperature and pH. The growth of aerobes in a SmF culture is greatly affected by the accessibility of substrates, energy (i.e., ATP), and DO. SmF is of a heterogeneous culture, whereby the reactions rates can be restricted by the MTR (i.e., substrates) at a particular interface. Various modes of operations such as batch, fed-batch, continuous, and semicontinuous are also possible in SmF for the improvement of TA lipases production in order to attain an optimal and cost-effective biomanufacturing process. SmF is also suitable for the production of lipases by filamentous fungi and many bacteria where the concentration of the carbon source (i.e., oil) and nitrogen source had a significant effect on lipase production [101]. For instance,* B. methylotrophicus* PS3,* S. pasteurii* COM-4A,* B. subtilis* COM-6B,* Aspergillus* sp. strain O-8,* T. lanuginosus* (i.e., GSLMBKU-10, GSLMBKU-13, and GSLMBKU-14), and* S. arlettae* JPBW-1 [8, 50, 62, 66]. Strain such as* Aspergillus* sp. strain O-8 lipase produced from the SmF has more stability at higher temperature than SSF [41, 62]. Lipase produced from SmF still retained about 72% of residual activity after one hour of incubation at 90°C. Furthermore, lipase produced from SmF retained 80% of the residual activity at the acidic pH while lipase obtained from the SSF results in residual activity of 60% at the alkaline pH [62]. In SmF, the highest lipase production was at 37°C and pH 7.2 while in SSF optimal temperature and pH are at 35°C and pH 6.0, respectively [41, 62].

Fed-batch culture has been applied for the production of lipases by* Acinetobacter radioresistens* and* Candida cylindracea* NRRL Y-17506, where Tween-80 and oleic acid were added intermittently and stepwise feeding to the initial batch fermentation after carbon sources supply was exhausted [24, 102]. However, lipases production may decrease at high specific growth rate in the later part of fed-batch cultures due to the build-up of carbon source oversupplied [102]. Several studies have showed that the growing cells and excessive carbon source may suppress the metabolic pathway of TA lipases production.

## 6. Fermentation Time

The TA lipases fermentation by many producing strains was classified as a growth-associated process. Growth and fermentation time normally reached the maximum TA lipases production after 24 h up to several days, depending on fermentation optimized condition, mode of operation, type of TA lipases produced, and strains. The TA lipase production was usually maximal when the fermentation reached a maximum growth or at the beginning of the stationary phase and then gradually decreased towards the end of batch fermentation [27]. For instance, a maximal lipase activity* Acinetobacter* sp. AU07 of 331.16 U (specific activity of 38.64 U/mg) was obtained in SmF at optimal temperature of 30°C and pH 7.0 for 16 h fermentation time when it reached its stationary growth phase [27]. The fermentation time to obtain maximum TA lipase activity by* Acinetobacter* sp. AU07 was similar to several TA lipase productions like by* Acinetobacter* sp. BK44 and* B. thermoleovorans* ID-1 where maximal lipase activity can be observed later than 12 h fermentation time [27, 28]. The TA lipase production by recombinant* Pichia pastoris* X33 harbouring lipase V3 gene can reach maximum lipase activity in fed-batch cultivation after 168 h induction with MeOH in STB [83, 91]. Meanwhile, TA lipase produced by* T. harzianum* IDM14D reached its maximum activity (0.24 U/mL) and biomass concentration (1.25 g/L) in SFC at 30°C for 7 d fermentation time [47]. Lipase production from* A. cinnamomea* BCRC 35396 was enhanced by 0.01% (v/v) olive oil as an inducer in an aerated STB to give final lipase activity of 26 U/ml after 18 d fermentation time [11]. Moreover, the fermentation time of TA lipase IIT-BT L139 was comparatively short (60 h) in STB as compared to SFC (70 h) [34, 46]. It has been reported that the lipase production starts when the carbon concentration (i.e., glucose) reduced significantly and cell growth almost ceased and had entered the stationary phase. After optimal fermentation time, there might be an accumulation of fatty acids produced, which has been reported to repress lipase production [34].

On the other hand, continuous culture can be an ideal fermentation process for the production of microbial biomass and other growth-associated process such as TA lipases. Continuous fermentation differs from batch where fermentation time is prolonged for certain period of time [41]. Continuous mode of operation is an open system which allows simultaneous addition of nutrients and withdrawal of fermentation broth at a constant rate where the culture is maintained at its exponential phase [41, 92, 103]. For instance, TA lipases of thermoalkalophilic* Bacillus* sp. strain MC7 and thermophilic* Bacillus* sp. strain IHI-91 can be produced in continuous mode of operation [103, 104]. In the case for* Bacillus* sp. strain IHI-91, lipase activity showed a maximum of 340 U/L/h at a dilution rate of 0.4 per hour where its productivity was increased up to 50% as compared to batch fermentation [103].

## 7. Optimization Approaches

Process optimisation can be performed using technique such as OFAT and statistical tools such as RSM and artificial neural network (ANN) [92, 99, 105]. ANN showed a superior method over RSM for data fitting and estimation capabilities in optimization study of lipase production by recombinant* E. coli* BL21 and* Geobacillus* sp. strain ARM [92, 106] Different experimental designs have been employed for lipase production study and optimizations such as central composite design (CCD), Plackett–Burman Factorial Design (PBFD) and Box–Behnken Design (BBD) [41, 99, 101]. The model was later optimized using algorithms such as evolution algorithms (EAs) and Levenberg-Marquardt (LM) algorithm [107]. The standard LM algorithm uses trial-and-error method to estimate the damping factor and is less reliable for large-scale inverse problems [107]. In recent decades, there has been a growing interest in optimization based on the principle of EAs (survival of the fittest) which includes Genetic Algorithm (GA), Differential Evolution (DE), Particle Swarm Optimization (PSO), Evolutionary Programming (EP), Evolution Strategies (ES), and Genetic Programming (GP) [105, 108–111]. EAs have turn out to be very popular as function optimizers, because they are easy to implement and show fair performance for a broad range of functions.

This EAs approach was rarely reported but recent studies showed that GA and PSO approaches experimentally improved the optimization of lipase production [109, 110]. The overall optimized fermentation conditions obtained via PSO approach (96.18 U/gds in 46 generations) have slightly better performance, convergence, and computational efficiency as compared to that of the GA approach (95.34 U/gds in 337 generations) [110]. In another study, the optimization results also indicate that the lipase activity was significantly improved, where PSO (133.57 U/gds in the 27th generation) performs better than GA (132.24 U/gds in the 320th generation), slightly with regard to optimized lipase activity and highly in respective to convergence rate [109]. Therefore, the PSO approach with the minimal parameters tuning is a practical tool for optimization of fermentation conditions of lipases production [110]. The simple structure associated with the effective memory capabilities of PSO has proven to be a superior approach over GA [109].

Alternatively, DE, another type of EAs, is a simple, robust, compact-structured, stochastic direct search approach whose functions based on concepts of the survival of the fittest and natural selection, a common concept of EAs [107]. DE is also better than GA for fine tuning in complex search spaces. DE coupled with RSM has been proven to be a useful approach for the optimization of lipase production by* R. oryzae* via SSF [105, 107]. The input space of the experimentally validated RSM-model was optimized using this novel DE approach in which the maximal lipase activity of 96.52 U/gds was observed under DE optimal values (temperature, 35.59°C; pH, 5.28; liquid to solid ratio, 1.5; incubation time, 4.8 d) [105]. In another study, a maximal lipase activity of 134.13 U/gds was obtained which was higher than using OFAT approach (36.28 U/gds) under DE-optimized values (DMSO, 25%; buffer, 40 mM; soaking time, 128.5 min; temperature, 35°C) and DE-control parameters (number of population, 20; generations, 50; crossover operator, 0.5; scaling factor, 0.25). The use of DE approach has improved the optimization capabilities and decision speed, resulting in an improved extracellular* R. oryzae* lipase yield (36.28 U/gds) as compared to the OFAT approach [108]. The developed model and its optimization are generic to biological world, hence, appearing to be practical for the design and scale-up process of the lipase production by* R. oryzae* via SSF [108].

## 8. Conclusion

Lipases which are stable at high temperature and resistant to alkaline pH are demanding in biodiesel, detergent, leather, textile, Kraft pulp, and many other industries. The discovery of TA lipases reduced wastes and increased efficiency in such applications. TA lipases are also suitable with combination of surfactant, detergents, chelating agent, and ions for optimum reaction in respective industry. The optimized fermentation process and strategy (i.e., microbes, carbon-nitrogen composition, minerals, temperature, pH, agitation rate, aeration rate, and DOT) are important factors for high production of commercial TA lipases in industry. The development of recombinant and mutant strains showed their advantages over WT strains in producing high amount and stable form of TA lipases. By using correct fermentation techniques and optimization approaches, TA lipases can be produced efficiently and economically.

## Figures and Tables

**Table 1:** Characteristics of TA lipases.

TA lipases	MW	*T* (°C)	pH	Stimulants	Inhibitors	Chemicals with tolerance	References
*Pseudomonas* sp. lipase	-	90	11	-	-	-	[21]
BSK-L lipase	-	30–60	8	Mn^2+^, K^+^, Zn^2+^, Fe^2+^, Ca^2+^	-	SURF, oxidants, detergents	[22]
*Microbacterium* sp. lipase	40.0	50	8	Tri-X-100, SDS	PMSF, EDTA, DTT, Tw-20, Tw-80	-	[23]
PS3 lipase	31.4	55	7	Tri-X-100, Mg^2+^ Ca^2+^	M+ (A), SDS, EDTA, CTAB, Tw-80, glycerol	Solvents (A)	[24]
*S. aureus* lipase	-	60	12	-	-	-	[25]
*S. pasteurii* lipase	56.0	50	9.0	-	-	-	[8]
*B. stearothermophilus* lipase	67.0	55	9–13	Mg^2+^	-	SURF, oxidants, nonpolar solvent	[26]
BCRC 35396 lipase	60.0	25–60	7–10	-	-	-	[11]
*Acinetobacter *AU07 lipase	45.0	50	8	-	PMSF, SDS, H_2_O_2_, Tw-80, TriX-100, EDTA, DTT	-	[27]
LipB lipase	57.0	96	8	Mn^2+^ Ca^2+^	PMSF, EDTA	-	[15]
LipA lipase	50.0	96	8	Mn^2+^ Ca^2+^	PMSF, EDTA	-	[15]
Lip 256 lipase	33.0	80	9	Na^+^, Fe^3+^, K^+^, Fe^2+^, Sr^2+^	Ca^2+^, Mg^2+^, Cu^2+^, Solvent (D)	Glycerol, MaCN, pyridine, urea	[28]
*T. thermophilus* lipase	39.0	40–70	9.5	Ca^2+^	Zn^2+^, Cu^2+^	-	[29]
RSJ-1 lipase	37.0	50	8-9	Ca^2+^, Na^+^, Mg^2+^, Ba^2+^	Cs^+^, K^+^, Co^2+^, Zn^2^	EDTA	[30]
ID-1 lipase	34.0	70–75	7.5	Ca^2+^, Zn^2+^	-	-	[31]
LBN2 lipase	33.0	60	10	-	-	-	[9]
*Cohnella *sp. A01 lipase	29.5	70	8.5	-	-	EDTA	[18]
4R lipase	21.9	80	9.0	Mg^2+^	M+ (B), PMSF, orlistat, OA, I^−^, EDTA, urea	-	[4]
Ht7 lipase	22.0	90	9	Ca^2+^ Co^2+^ and Zn^2+^	-	-	[32]
*T. atroviride* 676 lipase	-	65	3–8	-	-	Solvents (B)	[33]
AV-5 lipase	50.0	65	9	-	-	-	[34]
BTS-3 lipase	31.0	55–70	8–10.5	K^+^, Fe^3+^, Hg^2+^, Mg^2+^	Al^3+^, Co^2+^, Mn^2+,^ Zn^2+^	-	[35]
CCR11 lipase	11.0	60	9-10	Ca^2+^, Tri-X-100	PMSF, SDS, Tw-80, Tw-20, butanol	-	[36]
*T. lanuginosus* lipase ln1	33.0	60–70	8–12	Ca^2+^	Fe^2+^, Zn^2+^, K^+^, Ag^+^	-	[37]
*B. thermocatenulatus *lipase	16.0	60–70	7.5–8	-	-	-	[38]
HF544325 lipase	27.0	45	8	-	-	-	[39]
*S. arlettae* JPBW-1 lipase	45.0	30–90	7–12	Mn^2+^, Ca^2+^ and Hg^2+^	K^+^, Co^2+^, Fe^2+^	Solvents (C), Tw-80, Tw-40, EDTA	[16, 40]
*Bacillus sp.* A30-1 lipase	65.0	-	-	-	-	-	[41]
*Staphylococcus aureus*	25.0	52	11	Ca^2+^ and Tween-80	SDS	-	[42]
Lipase L2	43.0	70	9	Ca^2+^, K^+^, Na^+^, Mn^2+^	EDTA, PMSF, pepstatin A, BME, DTT	-	[17, 43]

*Note*. MW, molecular weight; T, optimal or favourable temperature of enzyme activity; OA, oleic acid; Tw-20, Tween-20; Tw-80, Tween-80; SURF, surfactants; BME, 2-mercaptoethanol; solvent (A): methanol, ethanol, acetone benzene chloroform xylene; solvent (B): benzene, xylene, n-hexane, methanol, ethanol, and toluene up to 30%; solvent (C): kerosene, n-dodecane, and hexane; solvent (D): acetone, MeOH, trichloromethane, petroleum ether, hexane, isopropanol, DTT, EDTA, polyhexamethylene biguanide, DMSO, benzene, Tri-X-100, Tw-20, Tw-80, SDS; M+ (A): Cu^2+^, Fe^2+^, Zn^2+^, and Co^2+^; M+ (B): Co^2+^, Cd^2+^, Hg^2+^, Cu^2+^, and Pb^2+^; I^−^, iodine.

**Table 2:** Industrial application of TA lipases.

	Fields	Process	Products	Microbial origin of lipases	References
Main applications	Renewable energy	Transesterification of oils/alcoholysis/methanolysis/interesterification	Biodiesel	*Geobacillus thermodenitrificans* AV-5, *Microbacterium *sp., lipZ01 expressed in *Pichia pastoris* GS11	[23, 34, 44–48]
	Laundry/ dishwashing	Hydrolysis of lipid/ester bonds	Biodetergent	*Brevibacterium halotolerans* PS4, *Talaromyces thermophilus, Bacillus stearothermophilus*, *Thermosyntropha lipolytica, Staphylococcus aureus* ALA1, *Bacillus methylotrophicus* PS3, *Staphylococcus aureus* SAL3, *Bacillus *sp. BSK-L, *Geobacillus zalihae*	[15, 19, 20, 22, 24–26, 39, 49–53]
	Leather	Hydrolyse grease or fat from leather	Biodegreasing agent	*Geobacillus thermoleovorans* DA2,* Staphylococcus aureus*, *Staphylococcus arlettae* JPBW-1	[42, 54, 55]

Other applications	Cosmetic	Esterification of fatty acids and other compounds	Esters, plasticizers, and lubricants	*Thermomyces lanuginosus, Rhizomucor miehei, Pseudomonas cepacia*, *Candia antarctica*	[5, 6, 56]
	Food	Transesterification/interesterification/acidolysis and esterification	AG, plasticizer, fatty acids, and esters	*Rhizomucor miehei*, *Rhizopus oryzae* NRRL 3562, *Candida antarctica*	[6, 16, 47, 57]
	Bioremediation	Hydrolysis of oils	Biodegrading agent	*Staphylococcus pasteuri, Ochrobactrum intermedium *strainMZV101, *Bacillus sonorensis* 4R, *Aspergillus ibericus, Aspergillus uvarum, Aspergillus niger*	[4, 41, 44, 58]
	Pharmaceutical	Transesterification/aminolysis	Plasticizer and drugs	*Candida antarctica, Candida rugosa*	[2, 6]

*Note*. AG, acylglycerols.

**Table 3:** TA lipases producing strains.

	Type	Microorganisms	Origin	References
Gram positive	Hyperthermophile	*B. sonorensis* 4R	Thar desert ecosystem	[4]
		*Bacillus *sp. HT19	Hot spring	[28]
	Thermophile	*Geobacillus thermoleovorans* DA2	desert/hot springs of Southern Sinai	[54]
		*Geobacillus thermoleovorans* ID-1	Water/soil of the thermal springs	[31]
		*Cohnella thermotolerans*	Alkaline Lonar Lake	[18]
		*Thermosyntropha lipolytica* DSM 11003	Alkaline hot springs of Lake Bogoria	[15, 39]
		*B. stearothermophilus*	Olive oil mill	[26]
		*Staphylococcus aureus*	Barbeque shop soil	[42]
	Mesophile	*S. aureus* ALA1	Dromedary milk	[49, 51]
		*Staphylococcus pasteuri*	Oil mill effluent	[8]
		*B. subtilis* BSK-L	Soil	[22]
		*Microbacterium *sp.	Sludge/sediment of Pulp and Paper Mill	[23]
		*Aeribacillus *sp. SSL096201	Lonar lake water	[59]
		*Bacillus* sp. LBN 2	Soil sample of hot spring	[9]
		*B. methylotrophicus* PS3	Soil of service station	[50]
		*B. licheniformis* Ht7	Soil of the Hayran thermal springs	[32]
		*B. pumilus* HF544325	Tannery waters in the old medina of Fez	[60]
		*Staphylococcus arlettae *JPBW-1	Rock salt mine	[55]

Gram negative	Mesophile	*Acinetobacter *sp.	Distillery waste	[27]
		*Pseudomonas *sp.	Local compost	[21]
		*Enterobacter* sp. Bn12	Soils/wastewaters from leather/edible oil industries	[61]
		*Ochrobactrum intermedium *MZV101	Gheynarje Nir hot spring	[58]

Fungus	Thermophile	*Talaromyces thermophilus*	Soil from thermal station	[20, 29]
		*Trichodermaatroviride* 676	Amazon forest soil	[33]
	Mesophile	*Curvularia *sp. DHE 5	Soil samples from Mit Ghamr	[57]
		*Antrodia cinnamomea* BCRC 35396	Taiwan Bioresources Collection	[11]
		*Aspergillus *sp. strain O8	Diesel contaminated soil	[62]

**Table 4:** The inoculums size, medium composition, temperature, pH, production duration time, and activity of TA lipases.

Strain/origin	IS (%)	Medium composition (%, w/v or v/v)	Temp (°C), agitation (rpm)	pH	Other minerals (%, w/v or v/v)	Duration (h)	Activity (U/mL or other units)	References
*B. stearothermophilus*	1	Xylose, 1; peptone, 1; olive oil, 1	55, 200	11	MgSO_4_; Tw-80	48	1800–2500	[26]
*Geobacillus *sp.DA2	4	Galactose, 1; (NH_4_)_3_PO_4_,, 0.5	60, 120	10	-	48	1000	[54]
*Bacillus *sp.ID-1	-	Olive oil, 1.5	65, -	-	-	12	520 (IU)	[31]
*C. thermotolerans*	5	Maltose, 1; starch, 1	60, 120	-	-	72	-	[18]
*B. methylotrophicus*	10	NB; tributyrin, 1	40, -	7	Ca^2+^; Tw-80, 1	72	360 (IU/mL)	[50]
*S. aureus* ALA1	-	Xylose, 1; YE, 1; olive oil, 1	30, -	8	-	30	128	[49, 51]
*S. pasteuri*	2	Coconut oil mill waste, 2	35, 120	7	-	48	20	[8]
*B. subtilis* BSK-L	1	YE, 0.5; BE, 0.5; peptone, 1	37, 200	7	NaCl, 0.2; olive oil, 1	24	120 (U/g)	[22]
*Microbacterium *sp.	-	Tributyrin, 1; NaNO_3_, 0.0085; Fe(CH_3_COO)_3_NH_4_, 0.005	37, -	8.5	MgSO_4_, 0.02; KH_2_PO_4_, 0.68; Na_2_HPO_4_·2H_2_O, 0.78	72	355 (U) or 3.2 (U/mg)	[23]
*Aeribacillus sp.* SSL	-	YE, 1; olive oil, 1	70, -	8	-	7 d	1.339 (*µ*M/min)	[59]
*Bacillus* sp. LBN 2	a	Groundnut oil, 1; peptone, 1	50, 200	9	NaCl, 7	48	20 (U/g)	[9]
*B. sonorensis* 4R	5	Tw-80, 1; glucose, 1; (NH_4_)SO_4_, 0.2	80, -	9	K_2_HPO_4_, MgSO_4_, NaCl, CaCO_3_, CaSO_4_, FeSO_4_, MnCl_2_, ZnCl_2_ (2 to 0.001)	96	177 (U/mg)	[4]
*B. licheniformis* Ht7	-	Olive oil, 1	30, -	-	-	24	15.9 (U/g)	[32]
*B. coagulans* BTS-3	-	Mustard oil, 1; peptone : YE, 1 : 1	55, -	8.5	-	48	1.16	[63]
*B. thermoleovorans*	1	NB, 0.325; olive oil, 2.5	55, 150	6.5	CaCl_2_, 0.1%; gum Arabic, 1	44	6000	[64]
*B. pumilus* HF544325	-	Casein peptone, 1.7; YE, 0.5	37, 200	7.4	Glucose, 0.25	72	14	[60]
*S. arlettae *JPBW-1	10	Soybean oil, 5	35, 100	8	-	3	-	[55]
*Pseudomonas sp*	2 b	Glucose, 1; NH_4_Cl, 0.5; ECO, 2; (NH_4_)_2_HPO_4_, 0.3	37, 250	7	K_2_HPO_4_, 0.3; KH_2_PO_4_, 0.1; MgSO_4_·7H_2_O, 0.01	24	24 (U/mg)	[21]
*O. intermedium*	-	YE, 0.5; olive oil, 1; NH_4_Cl, 0.1	60, 180	10	MgSO_4_·7H_2_O, FeSO_4_·7H_2_O, CaCl_2_·7H_2_O, K_2_HPO_4_ (1 to 0.1)	72	-	[65]
*T. thermophilus*	-	Tributyrin-olive oil emulsion, 1	50, -	9.5	-		7.3–10 (kU/mg)	[20, 29]
*T.atroviride* 676	1 c	Tw-80, 0.5; olive oil, 1; YE, 15	28, 105	6	(NH_4_)_2_SO_4_, 0.25; MgSO_4_, 0.2	4 d	175.20	[33]
*Curvularia *sp. DHE	d	Wheat bran, 1	30, -	7	Olive oil, 2; KCl, 0.05; MC, 70	7 d	83.4 (U/g)	[57]

*Note*. IS, inoculums size; NaCl, sodium chloride; K_2_HPO_4_, potassium hydrogen phosphate; KCl, potassium chloride; Na_2_S·9H_2_O, sodium sulphide; NH_4_Cl, ammonium chloride; MgCl_2_·6H_2_O, magnesium chloride hexahydrate; CaCl_2_·2H_2_O, calcium chloride dehydrate; NaHCO_3_, sodium bicarbonate; Na_2_CO_3_, sodium carbonate; C_3_H_7_NO_2_S, cysteine; (NH_4_)_3_PO_4_, ammonium phosphate; YE, yeast extract; BE, beef extract; a, 10^4^ cells/mL; ECO, emulsified coconut oil; b, 14 h old culture (1.3 × 10^9^ cell/ml); MC, moisture content; SSF, solid-state fermentation; c (10^6^ spores/ml); d (51.27 × 10^7^ spore/mL).

**Table 5:** The production of TA lipases via improved strains.

Gene of origin	Expression of lipase gene	Medium	*T*_*g*_, *T*_*p*_	Agitation (rpm)	pH	DOT (%), aeration rate (L/min)	Induction	Duration (h)	Activity	References
*Enterobacter* sp. Bn12	E. coli (BL21) pLysS and pET-26(+)	LB + antibiotics	37, 20	-	-	-, -	0.5 mM IPTG (OD_600_: 0.6)	18	2900 (U/mg)	[61]
*Thermomyces lanuginosus* (lipase gene ln1)	Pichia GS115 and expression plasmid vector pPIC9K (Heterologous expression under AOX1 promoter)	BMGY medium	28, 28	220	-	-, -	1.0% (v/v) MeOH daily (OD_600_: 2.0–4.0)	168	1328 (U/mL)	[66]
*Geobacillus thermocatenulatus*	*E. coli* BL321 carrying the PCYTEXP1 plasmid	Modified LB	30, 45	200 to 600	7.0	30, 3	Using temperature-inducible kPL promoter (OD_600_: 0.4–0.6)	12	770,000 (U/g DCW)	[67]

*Note*. *T*_*g*_, temperature for growth; *T*_*p*_, temperature for production; BMGY, buffered methanol complex medium; AOX1, alcohol oxidase 1 promoter; LB, Luria-Bertani medium; NaCl, sodium chloride; MeOH, methanol; modified LB medium containing 5 g/L NaCl, 10 g/L Bacto yeast extract, 10 g/L Bacto tryptone, 10 g/L glucose, 10 mM MgSO4, and 40 mM K2HPO4, together with 100 lg/mL of ampicillin; DCW, dry cell weight.

**Table 6:** The production of TA lipases in bioreactors.

Bioreactors (size)	Strain	IS (%)	Medium composition (%, v/v or w/v)	Temperature (°C), agitation (rpm)	Aeration rate (L/min), DOT (%)	pH	Other minerals/parameters	Duration (h)	Activity (U/mL)	References
3 L	*Acinetobacter* sp. AU07	0.5	Castor oil, 2	30, 150	-, -	7	-	12	48	[27]
2 L	*G. thermodenitrificans*	-	Waste cooking oil, 2	50, 400	1-2, -	8	-	-	330	[34]
2 L	*Antrodia cinnamomea* BCRC 35396	10	Glucose, 3; peptone, 0.5; yeast extract, 0.3; malt extract, 0.3	28, 150	2, -	4	KH_2_PO_4_, 0.1; olive oil, 0.01; vitamin B1, 0.1; MgSO_4_·7H_2_O, 0.1	18	26	[11]
20 L	*Bacillus* sp. RSJ-1	0.1	Peptone, 0.75; yeast extract, 0.75	50, 350	10, -	9.0	NaCl, 0.5; cotton seed oil, 0.75; Tween-80, 0.5; CaCl_2_·2H_2_O, 0.001	10	2.13 (U/mg)	[30]
1 L	*Citrobacter freundii* IIT-BT L139		Starch, 1 and peptone-urea, 1	40, -	-, -	9.0	-	60	12	[12]
14 L	*B. cepacia*	2	Glucose, 1; NH_4_Cl, 0.5; (NH_4_)_2_HPO_4_, 0.24	45, 300	4, 25	7	KH_2_PO_4_, 0.1; MgSO_4_·7H_2_O, 0.01; palm oil emulsified with gum acacia, <1	20	120 (U/mg)	[10]
20 and 100 L	*Thermosyntropha *DSM	-	Yeast extract, 0.75; NH_4_Cl, <1; cysteine, <1	60, -	-	8.2	NaCl, K_2_HPO_4_, Na_2_S·9H_2_O, Na_2_CO_3_, MgCl_2_·6H_2_O, KCl NaHCO_3_, CaCl_2_·2H_2_O (<1)	15–21	0.12–0.15 (U/mg)	[15, 39]

*Note*. STB, stirred tank bioreactor; IS, inoculums size; vvm, vessel volumes per minute; DOT, dissolved oxygen tension; NaCl, sodium chloride; K_2_HPO_4_, potassium hydrogen phosphate; KCl, potassium chloride; Na_2_S·9H_2_O, sodium sulphide nonahydrate; NH_4_Cl, ammonium chloride; MgCl_2_·6H_2_O, magnesium chloride hexahydrate; CaCl_2_·2H_2_O, calcium chloride dehydrate; NaHCO_3_, sodium bicarbonate; Na_2_CO_3_, sodium carbonate; C_3_H_7_NO_2_S, cysteine; (NH_4_)_3_PO_4_, ammonium phosphate; BE, beef extract; ECO, emulsified coconut oil.
